# Phospholipid signaling pathway in Capsicum chinense suspension cells as a key response to consortium infection

**DOI:** 10.1186/s12870-021-02830-z

**Published:** 2021-01-25

**Authors:** María E. Sánchez-Sandoval, Graciela E. Racagni Di-Palma, Victor M. González-Mendoza, Yahaira A. Cab-Guillén, José A. Muñoz-Sanchez, Ana Ramos-Díaz, S. M. Teresa Hernández-Sotomayor

**Affiliations:** 1 Unidad de Bioquímica y Biología Molecular de Plantas, Centro de Investigación Científica de Yucatán, Mérida, Yucatán Mexico; 2 Departamento de Biología Molecular, Universidad Nacional de Río Cuarto, Río Cuarto, Córdoba, Argentina; 3 CONA CYT- Centro de Investigación y Desarrollo en Agrobiotecnología Alimentaria (Consortium between Centro de Investigación y Desarrollo, A.C. and Centro de Investigación y Asistencia en Tecnología y Diseño del Estado de Jalisco), San Agustín Tlaxiaca, Hidalgo, Mexico; 4 Centro de Investigación y Asistencia en Tecnología y Diseño del Estado de Jalisco (CIATEJ), Subsede Sureste, Yucatán, Mexico

**Keywords:** *C. chinense*, *Colletotrichum* species, Phosphatidic acid, Plant-pathogen, Biochemical response, Phospholipase

## Abstract

**Background:**

Mexico is considered the diversification center for chili species, but these crops are susceptible to infection by pathogens such as *Colletotrichum* spp., which causes anthracnose disease and postharvest decay in general. Studies have been carried out with isolated strains of *Colletotrichum* in *Capsicum* plants; however, under growing conditions, microorganisms generally interact with others, resulting in an increase or decrease of their ability to infect the roots of *C. chinense* seedlings and thus, cause disease.

**Results:**

Morphological changes were evident 24 h after inoculation (hai) with the microbial consortium, which consisted primarily of *C. ignotum*. High levels of diacylglycerol pyrophosphate (DGPP) and phosphatidic acid (PA) were found around 6 hai. The*s*e metabolic changes could be correlated with high transcription levels of diacylglycerol-kinase (*CchDGK1* and *CchDG31*) at 3, 6 and 12 hai and also to pathogen gene markers, such as *CchPR1* and *CchPR5.*

**Conclusions:**

Our data constitute the first evidence for the phospholipids signalling events, specifically DGPP and PA participation in the phospholipase C/DGK (PI-PLC/DGK) pathway, in the response of *Capsicum* to the consortium, offering new insights on chilis’ defense responses to damping-off diseases.

**Supplementary Information:**

The online version contains supplementary material available at 10.1186/s12870-021-02830-z.

## Background

Chili was domesticated in Mexico, where 4 of the 5 species of *Capsicum* are cultivated, including *C. frutescens, C. annuum, C. pubescens* and *C. chinense*. However, *C. chinense* is the only species of chili thought to have originated in Mexico, given its uses, including traditional uses, among settlers from the Yucatan region. Under field conditions, these crops are susceptible to pathogen infections, among which special attention has been paid to *Colletotrichum* spp.

The *Colletotrichum* genus has been related to anthracnose disease and postharvest decay in a wide range of tropical, subtropical and temperate fruits, crops and ornamental plants [1–4].

Species, such as *C. acutatum, C. boninense*, *C. brevisporum*, *C. cairnsense, C. capsica, C. cliviae, C. coccodes, C. dematium, C. fructicola*, *C. queenslandicum, C. scovillei*, *C. siamense, C. simmondsii, C. truncatum,* and *C. gloeosporioides* have been detected in *Capsicum* spp. plants with anthracnose symptoms [5–7].

The mechanism of *Colletotrichum* infection and the defense mechanism of *Capsicum* have been reported from studies carried out with isolated *Colletotrichum* strains [8, 9].

During plant-pathogen interactions, a precise signaling process is indispensable for the successful adaptation and survival of the plant. There are many studies on host plant defense systems and pathogenic invasion effectors along with their hormone related pathways, such as salicylic acid (SA) and/or jasmonic acid (JA) [10–15]. However, there have been only a few studies related to the phospholipid signal transduction pathways associated with the response to pathogens, particularly the pathways that involve phospholipid-derived molecules as second messengers, or hypersensitive response (HR) [16, 17]. Phosphatidic acid is a very important signaling molecule that can modulate the activities of kinases, phosphatases, phospholipases and other proteins involved in membrane trafficking, calcium signaling and biotic and abiotic stress responses [18–20].

In a previous study, we evaluated the effect of SA and methyl jasmonate (MJ) on phospholipid signaling in *C. chinense* Jacq. cell suspension cultures. Treatment with SA inhibited phospholipase C (PLC) and phospholipase D (PLD) activities, while treatment with MJ increased both phospholipases activities [20]. Regarding the hypersensitive response, the PA content was related to the onset of HR and phospholipidic signalling in an over-activation of defensive response lines (Disease Suppression 1, DS1, from tobacco plants), which were challenged with *Ralstonia solanacearum* [17, 21]. Transient accumulation of both PA and diacylglycerol pyrophosphate (DGPP) studies were conducted in tomato suspension cells [22, 23]. Cells treated with a pathogenic elicitor showed high levels of PA, which was subsequently metabolized to DGPP [22]. When DGPP and PA were added, the induction of the expression of elicitor-responsive genes was observed in the absence of the elicitor [23].

The roots of plants establish a relationship with the microorganisms present in the rhizosphere. These interactions can involve either beneficial or pathogenic microorganisms [24, 25]. Both types of interactions trigger a complex response that determine the success of pathogenic proliferation and development in the plant. In recent years, the response of plant cells to pathogenic microorganisms has been studied mainly using single pathogen species [26–29]. However, the cellular response is even more complex under field conditions, since microorganisms tend to form consortia with compatible microbes [30]. These microbes may have an inhibitory or synergistic effect on the onset and development of an infection. Here, we propose to study the cellular and biochemical responses to the microbial consortium isolated from rotting roots and fruits in *C. chinense* plants.

In the present work, a *C. chinense* suspension cell system was used to study the role of the phospholipid signalling pathway in response to a consortium infection. We sought to first correlate the generation of important phospholipid-derived molecules with the expression of the associated genes in response to the microbial consortium from *C. chinense* plants in order to understand the phospholipid signal transduction that occurs during the interaction between *C. chinense* suspension cells and a microbial consortium, primarily consisting of *C. ignotum* (93.6%).

## Materials

Radiolabeled [^32^P] γ-ATP was obtained from Amersham Pharmacia Biotech (UK). The bicinchoninic acid (BCA) protein assay reagent was purchased from Pierce Chemical Co., Ltd., and other chemicals were provided by Sigma Aldrich. Murashige and Skoog (MS) medium were supplied by Phytotechnologies Inc. The commercial ZimoBiomics DNA kit was purchased from Zimo Research. Chloroform, methanol, pyridine, and formic acid were purchased from J.T. Baker Co. TLC plates were supplied by Merck®.

### Biological material

*Capsicum chinense* cell suspensions were obtained via the disaggregation of calli and cultivation in MS [31] at pH 5.6. This medium was supplemented with 0.5 mM myo-inositol, 0.02 mM thiamine, 0.2 mM cysteine, 4 μM 2,4-dichlorophenoxyacetic acid and 3% sucrose according to a previous report [20]. The cells were subcultured every 14 days as previously reported [20] and grown with shaking (100 rpm) under continuous light at 25 °C.

The consortium, was obtained from the rhizosphere of *C. chinense* plants with symptoms of wilt from Yucatán, México. For growing the microbial consortium, the method used, was described by Dhingra and Burton [32]. The consortium (mycelium) was maintained at 25 °C in the dark on modified agar media (2% Bacto agar, 10% vegetable juice (Herdez V8 juice, containing 8 vegetables, carrot, tomato, beetroot, spinach, kale/leaf cabbage, celery, parsley and lemon juice) to get reproductive structures [33, 34]. The microbial consortium was cultivated in Petri dishes; the material was flooded with water and scrapped using the end of a sterile glass slide. The resulting suspension was filtered through 50 μm Miracloth, as described by Sharma et al. (2015) [35] and incubated at 37 °C for 3 h, as suggested in Shipton (1985) [36].

Then, it was maintained at 4 °C, and filtered again prior to use [37]. The filtrate was referred to as a conidial suspension (cs, cells/mL). In order to standardize the amount of inoculum employed in the infection experiments, the number of *C. ignotum* conidia was quantified. All cs used were attenuated by heating at 95 °C for 10 min.

### Analysis of the consortium microbial profile by next-generation sequencing

DNA isolation from the microbial consortium was performed using the commercial ZimoBiomics DNA kit (Zimo Research). The quality of the extracted DNA was examined by agarose electrophoresis (1%) with ethidium bromide (0.01%) and visualized under UV light. DNA samples were sent for analysis to LABSERGEN (CINVESTAV), where nuclear ribosomal internal transcribed spacer (*ITS*) region or 16S amplicons were produced with 300,000 Paired End reads, and MiSeq sequences were generated. Bioinformatics analyses were carried out using readpipeline and MG_RAST [38]. The identification of the strains was conducted via massive BLAST [39] searches in MG_RAST [38], and the taxonomy of each strain was determined using the following databases: Encyclopedia of Life (http://www.eol.org/) [40], Global Catalog of Microorganisms (http://gcm.wfcc.info) [41], Integrated Taxonomic Information System (https://www.itis.gov/) [42] and Livemap (http://lifemap-ncbi.univ-lyon1.fr/) [43].

### Inoculation process

For the inoculation process, one gram of *C. chinense* cells (FW) were suspended in 25 mL of fresh MS medium and let to stand in the same cultivation conditions (at 25 °C at 100 rpm in continuous light) for 30 min prior to the cs addition. Inoculation was performed using different concentrations of the original cs (1 × 10^1^, 1 × 10^4^ or 1 × 10^8^) under sterile conditions. These concentrations were chosen based on previous experiments reported with *Colletotrichum* [44]. Cells were harvested after specific periods of time (hours after infection or hai) for the different analysis that were performed; i.e. at 12, 24, and 48 h for morphological characterization; 1, 3, 6 and 12 h for transcript abundance or 6 h for lipid quantification. The cs concentration used for the morphological characterization, gene expression analysis and lipid analysis was 1 × 10^4^ cells/mL.

### Epifluorescence analysis

Suspension cells of *C. chinense* were infected (or not) with the consortium and washed three times with phosphate buffer saline (PBS) at 0.1 M and pH 5.7, and the suspension was diluted 1:10 in PBS. The cells were stained with the following dyes: 1 μM **4′,6-diamidino-2-phenylindole dihydrochloride (DAPI) (Sigma) for** nuclei; 1.76 μM FM4–64 Dye (*N*-(3-triethylammoniumpropyl)-4-(6-(4-(diethylamino) phenyl) hexatrienyl) pyridinium dibromide) (Invitrogen™) for endoplasmic membrane; 10 μM CellMask™ Plasma Membrane Stain (Molecular Probes™) for plasma membrane, and 2 μM Calcofluor White Stain (WCF) (Fluka™) for the cell wall. After 30 min of incubation at room temperature, fluorescence was observed via epifluorescence microscopy (Axioplan, Zeiss, Germany).

### Cell fixation and scanning electron microscopy

For scanning electron microscopy (SEM), the MS medium was discarded, and *C. chinense* cells were washed with PBS (to eliminate any MS medium left) and incubated with 40% formaldehyde, 50% ethanol, 5% acetic acid, and 5% distilled water (FAA solution) for 72 h at 25 °C with gentle agitation every 3 h. The samples were washed with PBS to eliminate the FAA solution. The cells were dehydrated in ethanol solutions in a sequential gradient of 30, 50, 70, 96, and 100% ethanol for 12, 12, 3, 2 and 1 h, respectively. After the cells were fixed, the samples were dried to the critical point with liquid CO_2_ using a Sandri-795 critical point dryer (Tousimis), metalized with gold (Denton vacuum Desk II) and observed using SEM (JEOL JSM 6360LV).

### Cell viability assay

*C. chinense* suspension cells infected with the consortium were washed 3 times with phosphate buffer, resuspended in 1 mL of PBS and gently mixed. Then, a 3-(4,5-dimethylthiazol-2-yl)-2,5-diphenyltetrazolium bromide (MTT) [45] (Sigma) solution was added to a final concentration of 0.5 mg mL^− 1^, and the mixture was incubated at 25 °C for 8 h in the dark. This method is a colorimetric assay that can be quantified on the basis of absorbance measurements. The ability of viable cells with active metabolism to convert MTT into formazan salts as a precipitate inside the cells was evaluated. MTT was solubilized with 1.5 mL of methanol solution (50% final concentration), and cells were incubated with this solution at 60 °C for 30 min. Finally, the cells were centrifuged at 1500 x g for 5 min. The supernatant was recovered, washed 5 or 6 times with methanol and mixed to determine the absorbance at 570 nm.

### Protein extraction and quantification

Frozen *C. chinense* cells, either previously infected or not, were pulverized in liquid nitrogen and homogenized with solution A (50 mM HEPES, pH 7.2, 0.25 M sucrose, 5 mM KCl, and 1 mM EDTA) with protease inhibitors (1 μg mL^− 1^ leupeptin, 1 mM phenylmethylsulfonyl fluoride (PMSF) and 1 μg mL^− 1^ aprotinine) [46]. The extract was centrifuged at 20,000 x g for 30 min at 4 °C, and the supernatant was centrifuged at 105,000 x g for 1 h at 4 °C. The obtained precipitate (membrane fraction) was resuspended in 200 μL of 50 mM HEPES, pH 7.4. The protein concentration in the extracts was determined with a modified bicinchoninic acid protein assay reagent [47], using bovine serum albumin (BSA) as a standard. The same conditions were used for protein extraction and quantification of the cs.

### Lipid kinase activity

The activity of lipid kinases was determined on the basis of [^32^P] γ-ATP incorporation into the corresponding endogenous substrate [48]. The phosphorylation assay was performed as reported by Racagni-Di Palma et al., [47] with minor modifications, using a reaction mixture with 50 mM HEPES at pH 7.4, 1 mM EDTA, 10 mM MgCl_2_, 1 mM ATP, 0.2 mM sodium vanadate, 0.5 mM DTT, [^32^P] γ-ATP (370 MBq) and 80 μg of membrane fraction proteins. The mixture was incubated for 2 min at 30 °C, and the reaction was finally stopped with 1.5 mL of chloroform:methanol (1:2, v/v).

### Lipid extraction and separation

Lipids were extracted and subjected to alkaline TLC to separate the different phospholipid species. Lipid extraction was conducted as described previously by Racagni-Di Palma et al. [46]. In each sample, 0.5 mL of 2.4 N HCl and 0.5 mL of chloroform were added, and the bottom phase was then carefully extracted and mixed with 2 mL of 1 N methanol: HCl (1:1, v/v). The lipids were dried under vacuum and resuspended in 200 μL of chloroform:methanol (9:1, v/v). Finally, the lipids were analyzed using thin layer chromatography (TLC) plates impregnated with solution I [1% potassium oxalate, 2 mM EDTA, and methanol:water (2:3, v/v)] and activated for 40 min at 110 °C. The plates were developed with solutions of chloroform:methanol:acetone:acetic acid:water (40:15:14:12:7, v/v) and chloroform:pyridine:formic acid (35:30:7, v/v) for the first and second dimensions of TLC, respectively [46]. The positions of the radiolabeled lipids were determined by autoradiography.

### Phylogenetic analysis

Phylogenetic testing was conducted on DGK proteins using complete amino acid sequences obtained from the SOL Genomics Network database (https://www.sgn.cornell.edu/) [49]. The sequences were aligned using ClustalW [50] and displayed with MEGA 6 [51] software, and the maximum likelihood method was employed with a robustness of 1000 bootstrap replicates. The *C. chinense* DGK homologs *DGK1*, *DGK2, DGK3, DGK5, DGK5L, DGK6* and *DGK7* were tested against the predicted proteins from tomato (*S. lycopersicum* ITAG release 2.4), coffee (*C. canephora* v1.0) and *Arabidopsis* to obtain their phylogenetic relationships.

### Gene expression assay and data analysis

Cultured cells of *C. chinense* were infected for 1, 3, 6 and 12 h with 1 × 10^4^ cs, and the consortium was analyzed. For the expression analysis, RNA was isolated using TRIZol™ RNA Reagent (Invitrogen™), and cDNA was synthesized using 500 ng of the total RNA with Revert Aid Reverse Transcriptase (Thermo Scientific). For the reverse transcription-quantitative polymerase chain reaction (RT-qPCR) assays, amplification was conducted using Maxima SYBR Green/ROX qPCR Master Mix (Thermo Scientific) and a PikoReal 24 real-time PCR system (Thermo Fisher Scientific, Ratatsie 2, FI-01620 Vantaa, Finland). The conditions for RT-qPCR were as follows: 1) initial denaturation step at 95 °C for 10 min; 2) two-step cycling at 95 °C for 40 s and Tm for 40 s with 40 or 45 cycles for each gene; and 3) final melting curve step from 56 °C to 95 °C. The primers used were designed based on pepper genome sequences (*C. annuum* cv. CM334 genome CDS) and tested in *C. chinense* as *CchDGK1, CchDGK3, CchNPC6, CchPR1a, CchPR5, CchTUBa* and *CchEF2a3L*, respectively (Table S1). Finally, for fold change determination, a 2^−ΔΔ CT^ method with an individual efficiency corrected calculation was used [52], *CchTUBa* and *CchEF2a3L* were used as a reference genes.

## Results

### Analysis of the consortium microbial profile

The isolated consortium from the rotten roots of *C. chinense* seedlings was characterized by NGS to identify the microorganisms present. The metadata were stored in the NCBI database with the registration number ID PRJNA479448. The bioinformatics analysis of the sequences enabled the identification of the microorganisms present in this consortium, with *C. ignotum* accounting for most of the eukaryotic microorganisms present (Fig. S1), while the predominant genus of prokaryotes was *Bacteroides* (Fig. S2), such as *Barnesiella sp.,Alistipessp, Pantoea sp., Acinetobacter sp., Parabacteroides, Kluyveromyces marxianus, Galactomyces geotrichum, Glomus sp., Exophiala sp., Malassezia restricta, Postia placenta, Elmerina caryae, Faecalibacterium, Clostridium IV, Halanaerobium, Veillonella, Phascolarctobacterium, Phascolarctobacterium, Lachnospiracea, Roseburia, Desilfonatronovibrio, Streptococcus*, (less than 3%; S1) The low proportion of reads obtained from 16S compared to those obtained from the ITS amplicon does not limit their relationship with the plant or with the majority strain. Thus, we consider the set of identified microorganisms as a consortium.

### Infection establishment

To understand the relationship between host and pathogen, *C. chinense* suspension cells were inoculated with the microbial consortium primarily consisting of *C. ignotum*. First, we started by determining the infection conditions and sampling times that resulted in a contrasting response at the morphological level, growth rate and/or exacerbation of death and could therefore provide more information regarding the topic investigated.

**Fig. 1:**
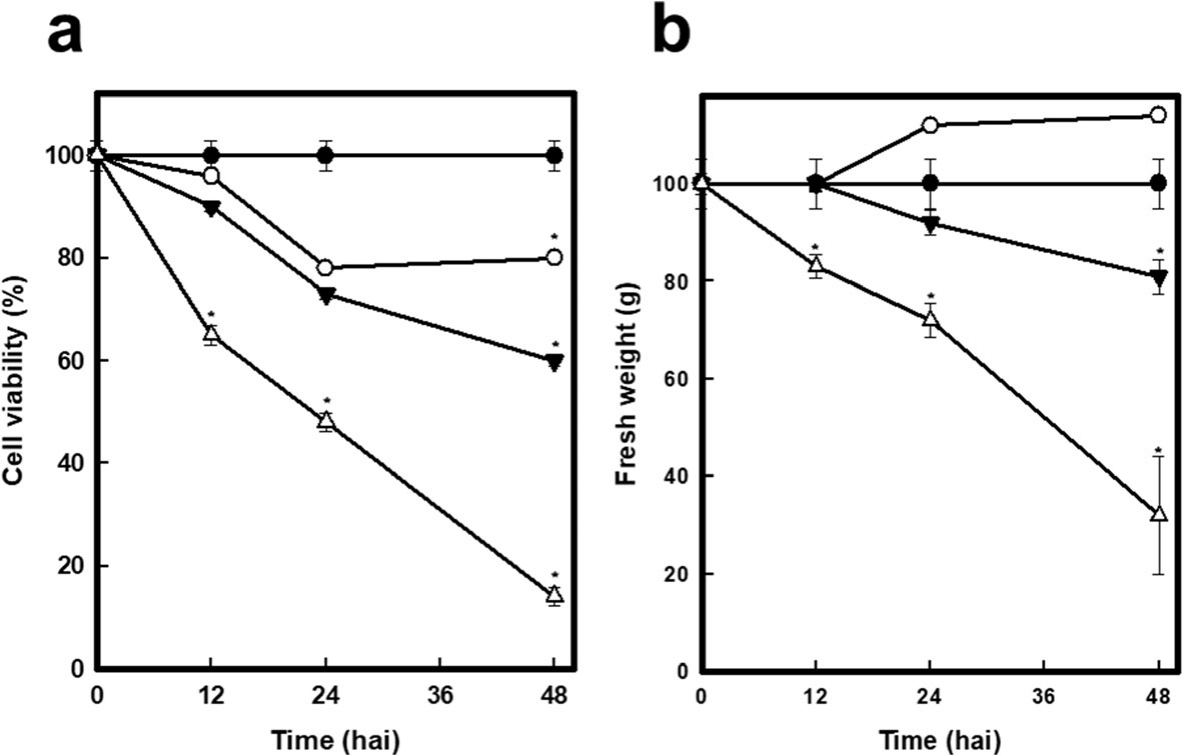
Evaluation of the optimal infection time using *C. chinense* cells infected with cs. **a**) Cells were infected with different amounts of cs (1 × 10^8^ = white triangles, 1 × 10^4^ = black triangles, 1 × 10^1^ = white circles) from the consortium and incubated for different times (hours after infection, hai). Cells without treatment are indicated in black circles. Cell viability was determined using MTT. **b**) Fresh weight of the cells after the infection treatments. Values are the means of three experiments with triplicates +/− SE, *p < 0.05

*C. chinense* cells that were infected with a 1 × 10^8^ cs (empty triangles) showed a decrease in viability of approximately 35% after 12 h and decreases of up to 50 and 90% at 24 and 48 h after inoculation, respectively (Fig. 1 a). Under treatment with a 1 × 10^4^ cs (filled triangles), decreases of approximately 15, 30 and 40% were observed at 12, 24 and 48 h after inoculation, respectively, whereas when cells were inoculated with a 1 × 10^1^ cs (open circles), a clear trend was not obtained (Fig. 1 a). The decrease in viability was also correlated with a decrease in fresh weight (Fig. 1 b). When the cells were exposed to the most concentrated cs, a greater decrease in viability as well as in fresh weight were observed (1 × 10^8^ cs). Finally, a 1 × 10^4^ cs allowed us to generate slight (30%) and/or severe cell damage (50%) after 24 and 48 h of treatment, respectively.

**Fig. 2:**
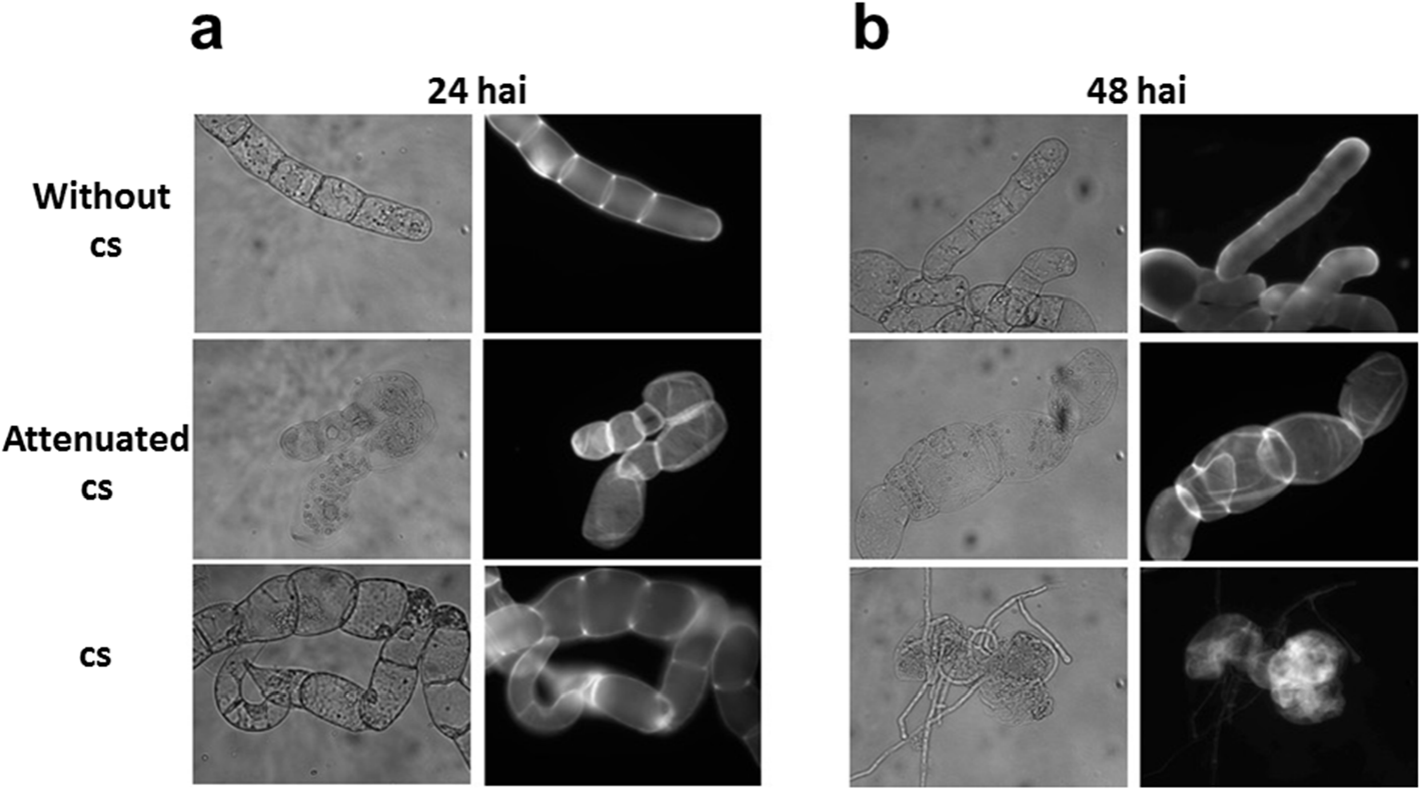
Morphological structure of *C. chinense* cells after infection with a cs. The cells (1 g) were treated for 24 (**a**) and 48 hai (**b**) without the cs, with the attenuated cs and with the unattenuated cs (1 × 10^4^), then stained with WCF and visualized using epifluorescence microscopy. The figures are representative of three independent experiments, with two images from each obtained via microscopy

**Fig. 3:**
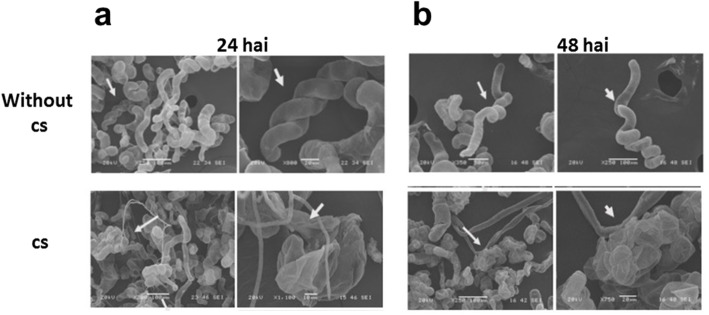
Morphological effects of cs treatment. The cells were treated for 24 (**a**) and 48 hai (**b**) without or with the cs (1 × 10^4^). The cells were observed using SEM. The figures are representative of three independent experiments with two images from each obtained via SEM (white arrows are pointing to *C. chinense* cells)

Images of *C. chinense* suspension cells in the presence of the microbial consortium showed an expanded cell phenotype after 24 h and a clearer phenotype at 48 h after inoculation compared to the control cells (Figs. 2, 3). Interestingly, when a viable consortium was compared with the attenuated version, these two consortia exhibited the same response to a blow-up cell phenotype (Fig. 2). Finally, at 48 h after the inoculation of a 1 × 10^8^ cs, the cell culture presented a dark brown color and null viability (data not shown).

With respect to the pathogens, the consortium showed a notable amount of growth after 12 h; major changes in hyphal abundance were observed at 48 h after inoculation when a 1 × 10^4^ cs was used (Figs. 2, 3) and increased hyphal abundance was observed when a 1 × 10^8^ cs was used (data not shown).

### Morphological changes during infection

*C. chinense* suspension cell morphology was evaluated after the cells were inoculated after 24 and 48 h with a 1 × 10^4^ cs from the microbial consortium. At 24 h after inoculation, the cells showed changes such as increased cell turgidity and a low abundance of hyphae in *C. chinense* cells and the microbial consortium, respectively (Fig. 3). Some *C. chinense* cells began to exhibit disruption when inoculated with a 1 × 10^8^ cs (6 h after treatment, data not shown).

**Fig. 4:**
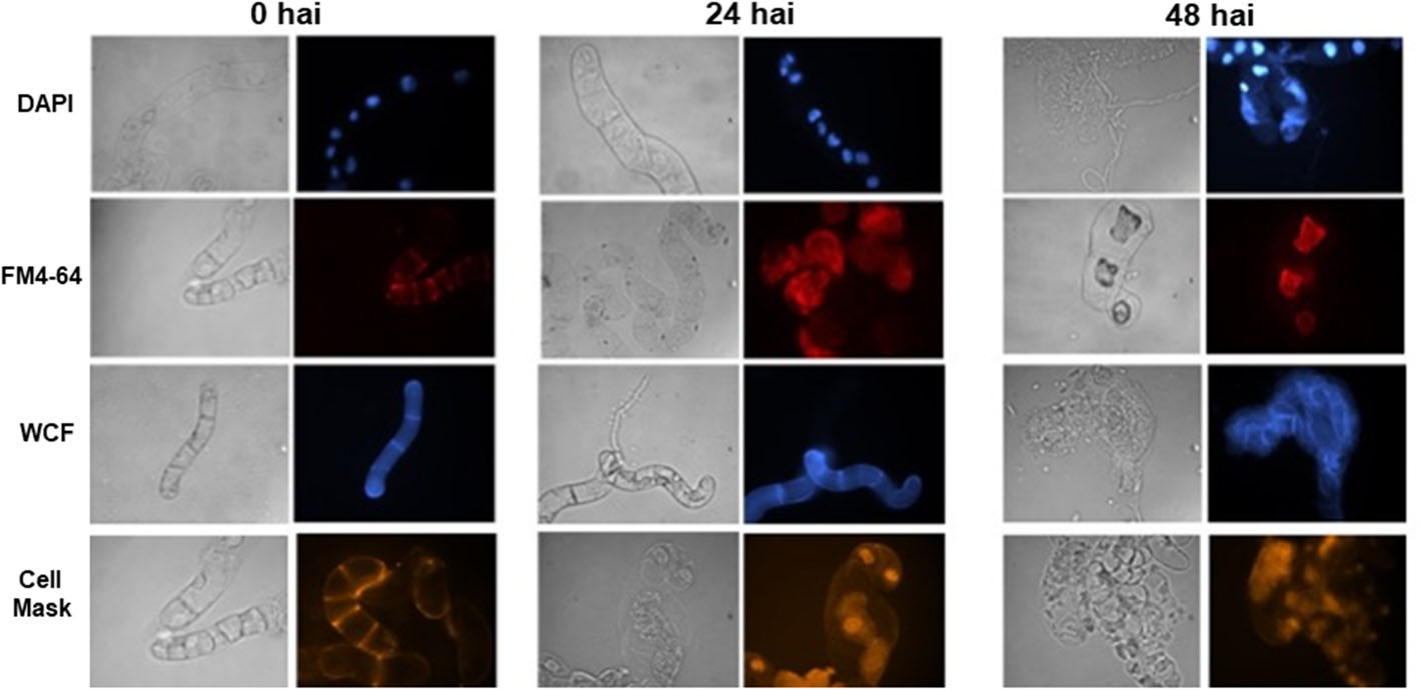
Cell integrity damage evaluation. The cells were treated with the cs as indicated above for 0, 24 or 48 hai. The left column shows cells with a visible field. The cells are stained with DAPI (nuclei in blue), FM4–64 (endoplasmic membranes in red), WCF (cell wall) and CellMask (cytoplasmic membrane). The figure is representative of three independent experiments

On the other hand, the cells were evaluated using fluorophores to observe the cell wall (WCF), cytoplasmic membrane (CellMask), endoplasmic membrane (FM4–64) and DNA integrity (DAPI; Fig. 4). During the experiments without the consortium, the structure of the *C. chinense* cells remained unchanged even 24 to 48 h after mock inoculation (non treated cells) (MS medium) (Fig. 4). When the *C. chinense* cells were inoculated, they showed damage to the plasma and endoplasmic reticulum membranes even 12 h after inoculation (Fig. 4). However, at 48 h after inoculation, the damage to both membranes (plasma and endoplasmic) and the cell wall was severe (Fig. 4).

The evaluation of DNA integrity with DAPI showed that prior to 24 h after inoculation, the cells accumulated moderate DNA damage, and DNA aggregation and fragmentation were observed at 48 h (Fig. 4).

### Changes in lipid kinase activity are involved in infection events

**Fig. 5:**
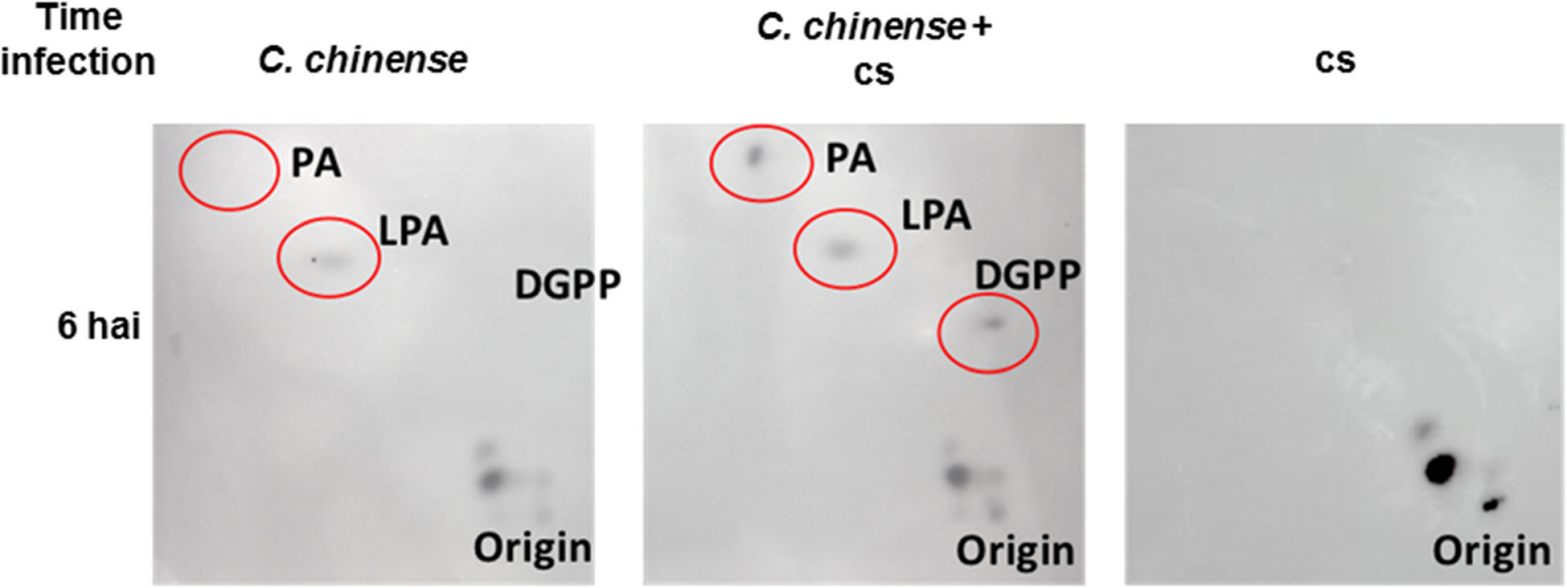
Detection of lipids produced by kinase activity using 2D-TLC. Lipids from *C. chinense* cell cultures infected for 6 h with a cs (1 × 10^4^) (*C. chinense* + cs) were subjected to alkaline TLC to separate the different phospholipid species. As a control, lipids were developed from only *C. chinense* cells or only the cs. Radioactivity was detected by autoradiography. A representative result from three experiments is presented

The effect of the microbial consortium on phospholipid-derived molecules in *C. chinense* cells was evaluated. Cells were incubated for 6 h in the presence or absence of the consortium (1 × 10^4^ cs). Lipid kinase activities were assayed. We observed that at 6 hai, the levels of PA, lysophosphatidic acid (LPA) and DGPP were higher in inoculated cells than in untreated cells (Fig. 5).

Next, the transcription profiles of DGK genes during infection events were analyzed and a phylogenetic test was conducted. In an in silico search, at least 7 DGK homologs (*CanDGK1, CanDGK2, CanDGK3, CanDGK6, CanDGK6-L, CanDGK6-L2* and *CanDGK7*) were found in the *C. annuum* genome (*C. annuum* cv CM334 genome CDS). These well annotated sequences were employed to guarantee that the designed primers would be used to find functional genes (Fig. S3).

Expression levels of genes for DGK homologs (*CchDGK1* and *CchDGK3*), a nonspecific PLC (*CchNPC6*) and also genes related to pathogenesis, such as PR (*CchPR1a* and *CchPR5*) were monitored at several infection stages up to 12 hai, given that at this time, the cells showed morphological changes without exhibiting extensive damage or cell death. *CchDGK1* and *CchDGK3* genes showed maximal increases in transcript abundance within the first hour after inoculation, and they maintained augmented expression for up to 12 hai when *C. chinense* suspension cells were challenged with a 1 × 10^4^ cs (Fig. 7). In non-treated cells, both *CchDGK1* and *CchDGK3* showed an important repression after 1 hai (Fig. 7). Regarding the pathogenesis related genes, both *CchPR1a* and *CchPR5* genes, were repressed after 1 hai. In addition, both genes showed augmented expression up to 12 hai, but in lower levels than the DKG homologs (Fig. 8). *CchPR1a* and *CcPR5* in non-treated cells showed a minor repression during the first 12 hai (Fig. 8). It is noteworthy to mention that *CchNPC6* also presented a moderated increase after 1 hai, to decrease subsequently (Fig. 8).

The phylogenetic assays for DGK from plants showed 3 well-defined groups. The first one included DGK1 and DGK2 (Fig. S3, in blue); the second included DGK3 and DGK7 (Fig. S3, in green); and DGK6, DGK6-L and DGK6-L2 were in the final group (Fig. S3, in red).

## Discussion

A plethora of mechanisms of interaction between plants and pathogenic microorganisms has been reported by several authors; however, to reproduce those interactions that occur in the field in our study model, we had to consider that the roots of plants interact with consortia of microorganisms to obtain a broader view of plant-microbiome relationships. In the present work, we isolated a consortium, which was then characterized by metagenomic analysis and applied to a cellular model of *C. chinense*, allowing us to observe cell signaling activated by this interaction. Metagenomics revealed that the major microorganism present was *Colletotrichum ignotum*, which is related to anthracnose disease in fruits [53].

In the in vitro infection system established in this study, all *C. chinense* cells grown in suspension exhibit the same probability of infection. Defined amounts of cs can be added to a standardized amount of *C. chinense* cells, and the infection can be followed by microscopy at different times (Figs.2, 3 and 4). Shortly after inoculation, primary infection was observed with the penetration of the hyphae into the cells and effects due to infection, such as the deterioration of the cell wall and a collapse of the plasma membrane followed by a stage in which fragmentation of the nuclei, can be observed (Figs. 3 and 4). The disruption of cells could be caused by programmed cell death in response to inoculation with the consortium. In contrast, after 48 h of inoculation, a complete reversal of the ratio of the plant cell vs hyphae populations occurred, and *C. chinense* cells died when the microbial consortium reached the largest hyphal population (Fig. 3).

Damage to the cell wall may result from the secretion of cellulases and pectinases that degrade the cell wall by the cs which, along with proteases, facilitates the initial penetration and infection of the host. In 2004 [54], Kim and collaborators observed nuclear changes and structural changes related to an hypersensitivity response in the fruit of *Capsicum annuum cv. Jejujaerae* (susceptible) and *Capsicum baccatum* cv. PBC80 (resistant) inoculated with the anthracnose pathogen *Colletotrichum gloeosporioides*, where degradation of the cell wall by enzymes secreted by the pathogen was observed. In addition, the separation of the plasma membrane from the cell wall, the swelling of the endoplasmic reticulum, the accumulation of dense inclusions in the vacuoles and cytoplasmic vacuolization accompanying fragmentation of the cytoplasm and DNA fragmentation were observed. Therefore, our results suggest that cell wall damage is a characteristic of pathogen attack (cs) in the cell suspension model of *C. chinense*.

Naton and colleagues [55] observed a reduction in cell viability and changes in the morphology of cell suspensions of parsley during infection with *Phytophthora infestans*. These changes in cells during infection are due to the formation of reactive oxygen species, particularly the highly aggressive oxygen radicals that produce lipid peroxidation [56, 57]. The effect of cs in *C. chinense* generated an increase in cell death that became evident over time.

In this study, the structural damage observed in the cells of *C. chinense* (Figs. 3 and 4) could be derived from various biochemical events occurring primarily in the plasma membrane. For example, unsaturated fatty acids can be oxidized and eliminated from the lipid bilayer since ROS (H_2_O_2_), which are generated as an initial response of cells to attack by a pathogen, can trigger the activation of lipoxygenases [57, 58].

In plants, PA and DGPP are well accepted as second messengers in signaling pathways and respond to biotic and abiotic stress [19, 22, 59–62]. As mentioned before, high levels of PA were found when *C. chinense* suspension cells were inoculated with a microbial consortium that primarily consisted of *C. ignotum*. Many authors have reported that PA is subsequently metabolized to DGPP in response to many types of biotic or abiotic stress, such as pathogens [21] water deficits [59], fungal elicitors [22], osmotic stress [63], Nod factors [64], and salt stress [65].

**Fig. 6:**
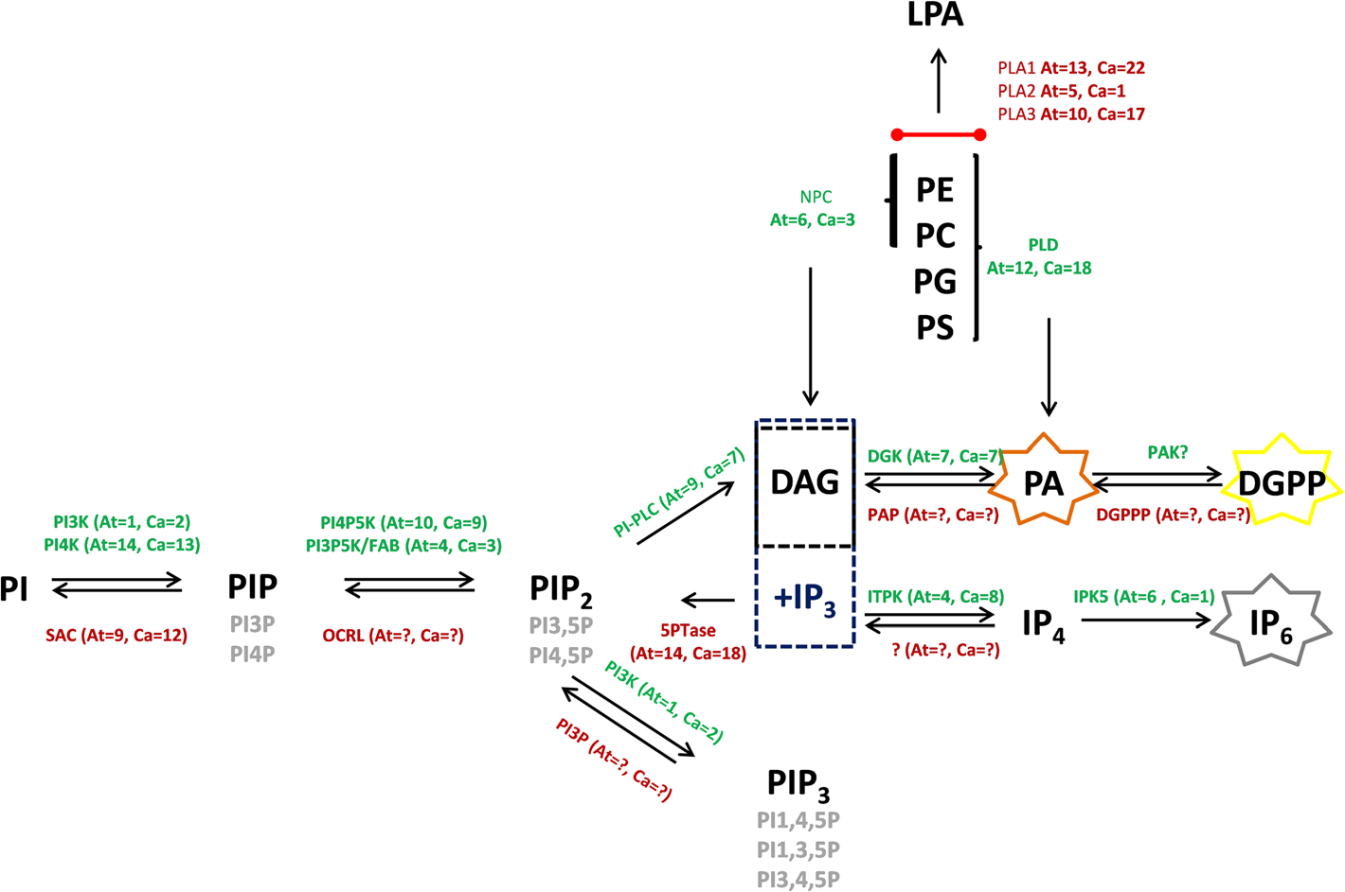
Production of PA and DGPP by different pathways and interconversion reactions mediated by phosphorylation and dephosphorylation. Green denotes phosphorylation reactions, and red denotes dephosphorylation reactions. Other colors indicate key enzymes in these pathways. In the phosphorylation pathway: PI3K or PI4K, phosphatidylinositol 3-kinase or 4-kinase, respectively; PI4P5K, phosphatidylinositol 4-phosphate 5-kinase; PI3P5K, phosphatidylinositol 3-phosphate 5-kinase; ITPK, inositol-tetrakisphosphate 1-kinase; IPK5, inositol-pentakisphosphate 2-kinase and DGK, diacylglycerol kinase. In the dephosphorylation pathway: PAP, phosphatidic acid phosphatase; 5PTase, inositol polyphosphate 5-phosphatase; PI3P phosphatidylinositol 3-phosphatase and PI5P, phosphatidylinositol 5-phosphatase. Key enzymes: PLD, phospholipase D; NPC, nonspecific phospholipase C; PI-PLC, phosphatidylinositol-specific phospholipase C and PLA, phospholipase A. PI, phosphatidylinositol; PIP, phosphatidylinositol phosphate; PIP_2_, phosphatidylinositol bisphosphate; PIP_3_, phosphatidylinositol trisphosphate; DAG, diacylglycerol; PA, phosphatidic acid; DGPP, diacylglycerol pyrophosphate; IPx, inositol polyphosphates; PC, phosphatidylcholine; PE, phosphatidylethanolamine; PG, phosphatidylglycerol and PS, phosphatidylserine

In plants, the phosphorylated forms of PA and DGPP have started to gain importance as signaling molecules involved in many stress responses [66]. Here, we report the presumptive activity of the PI-PLC/DGK phospholipid pathways and the phospholipid-derived molecules resulting from PIP or PIP_2_ hydrolysis forming second messengers, such as PA and DGPP, which eventually invoke downstream signaling responses to infection. In general, the detected PA could be obtained via two different pathways: one involving PLD, which generates PA directly by hydrolyzing structural phospholipids such as phosphatidylcholine (PC), while the other involves PI-PLC, which generates DAG, and DAG is subsequently phosphorylated to PA via the action of PLC/DGK [67] (Fig. 6). However, increasing evidence points to PA accumulation in relation to PI-PLC/DGK activity in response to pathogen effectors such as bacterial elicitors [22, 68], specific effectors from *Pseudomonas syringae* [69] and fungi, such as *Cladosporium fulvum* [67] and *Botrytis cinerea* [70]. In this manner, perhaps PA resulting from PLC/DGK activity could be produced via PIP conversion in the first stages of infection (6 h after inoculation).

PA may resulted in PIP_2_ conversion by PI-PLC activity, either via the direct hydrolysis of PIP_2_ or the initial transformation of PIP to PIP_2_ via the activity of a phosphatidylinositol 4-phosphate 5 kinase (PIP5K) (Fig. 6) [71–73]. However, the levels of LPA (Fig. 5) did not show significant changes compared to those in control cells, and/or minor changes could be generated by phospholipase A (PLA) activity through a route that may involve the turnover of PA (Fig. 6). On the basis of all of these data, we hypothesized that the increase in PA resulted in coordinated action between the PI-PLC/DGK pathways.

**Fig. 7:**
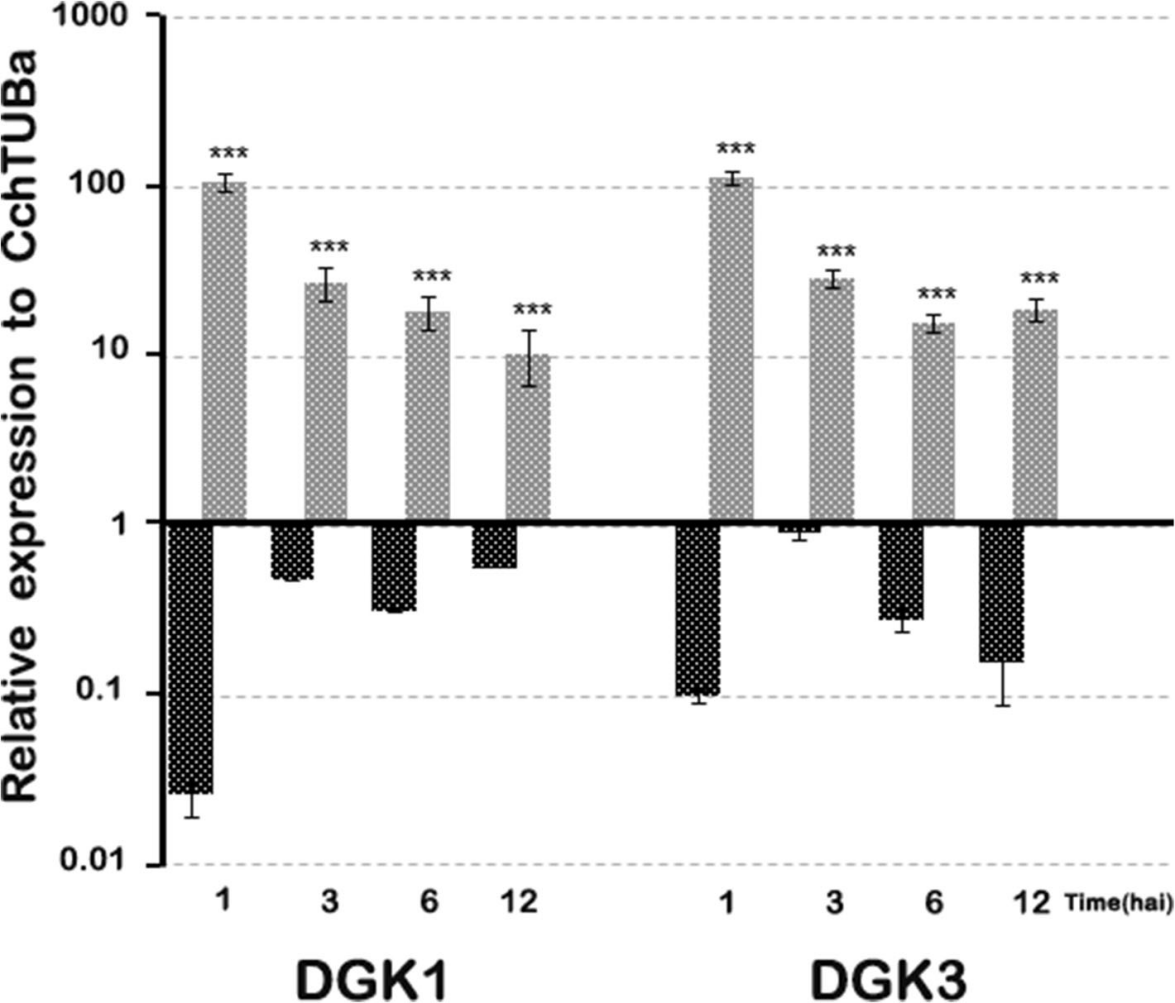
*CchDGK* transcription level during cs infection events. *C. chinense* cells were infected with a 1 × 10^4^ cs for different time periods (hai), RNA was extracted, and the relative expression of *CchDGK1* and *CchDGK3* was analyzed through real-time quantitative PCR. The gene expression was calculated taken as a reference gene *CchTUBa and CchEF2a3L,* (black bar) non treated cells, (grey bar) cells plus cs. Data from three independent experiments with three biological samples run in duplicate are presented as the mean +/− SE, ***p < 0.001

**Fig. 8:**
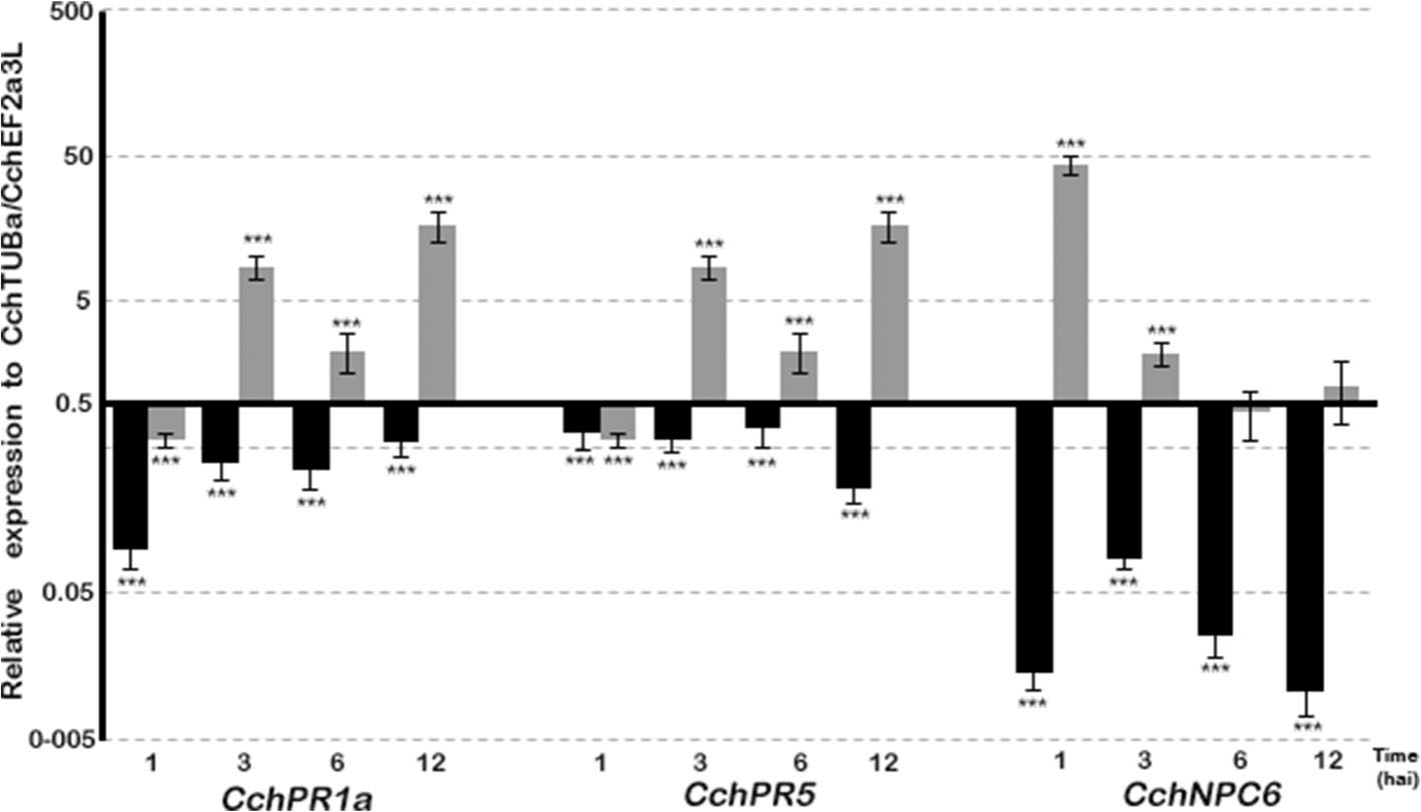
Pathogenesis-related genes and *NPC6* transcription levels during cs infection events. *C. chinense* cells were infected with a 1 × 10^4^ cs for different time periods, after which RNA was extracted, and the relative expression of pathogenesis-related transcripts (*CchPR1a* and *CchPR5*) and *CchNPC6* was analyzed through RT-qPCR taken as a reference gene *CchTUBa and CchEF2a3L*, (black bar) non treated cells, (grey bar) cells plus cs. Data from three independent experiments with three biological samples run in duplicate are presented as the mean +/− SE; ***p < 0.001

When these biochemical changes were contrasted to the DGK transcription profile during infection events, a correlation between PA or DGPP accumulation and the specific expression of DGK (*CchDGK1* and *CchDGK3*) was obtained, in which the results showed the highest accumulation of transcripts after 1 to 12 h of inoculation (Fig. 7) and were consistent with the analysis of marker genes related to pathogenesis (*CchPR1a* and *CchPR5*, Fig. 8), when *C. chinense* suspension cells were challenged with the microbial consortium. These results support the hypothesis that higher DGK transcript accumulation could be related to PA-DGPP levels in infection events between *C. chinense* suspension cells and the microbial consortium. These data support the notion that higher levels of PA can be produced by the activity of DGK in the phosphoinositide pathway in *C. chinense* suspension cells in response to infection events. Based on the results (activity and gene expression), it is probable that DGK seems to be involved in the signaling events of this pathogen. However, we would not exclude the possibility that the other enzymes (and genes) shown in Fig. 6 are also involved in this process. Furthermore, three PLCs and four PLDs were also analyzed in this study, and we did not observe an increase in gene expression at the times tested (data not shown).

However, expression of a gene for a non-specific PLC, such as *CchNPC6*, exhibited an important increase only at very short times (at 1 hai) and basal levels thereon (Fig. 8), probably pointing to a generalized response to cell culture manipulation. In contrast, DGKs expression maintained higher levels after 3, 6 and 12 h following inoculation, whereas *CchNPC6* was greatly reduced at those same times (Fig. 7). Pathogenesis-related genes, specifically *CchPR5,* presented a similar behavior to that of DGKs, increasing within the first hour to be reduced at 3 or 6 h and then increase again (12 hai, Fig. 8). This pattern demonstrates that the response observed in the beginning corresponds to a generalized response, while at 12 h, it changed to a specific response to the microbial consortium (Fig. 8).

Recently, Gonorazky et al. [70] demonstrated that the PLC/DGK pathway is required to regulate defense responses to the necrotrophic pathogen *B. cinerea* by transiently silencing *SlPLC2* in tomato plants. Zhang et al. [74] reported that the overexpression of rice DGK in tobacco enhances resistance to *Phytophthora parasitica* var. *nicotianae* and that the increase in the accumulation of PA confers disease resistance.

## Conclusions

This study demonstrated the activation of the phospholipid signaling pathway in response to a microbial consortium. Also, expression of DGKs, as well as that of some others related to pathogenesis, was affected in the presence of the consortium. The presence of the consortium also induced membrane disruption and important reduction in cellular viability. The next step in the research will be the use of phospholipases and kinases inhibitors to distinguish the contribution of each pathway to the microbial consortium response. The study of transcriptomics to detect gene clusters activated differentially by the microbial consortium and by other activation pathways of the phospholipid signal cascade is also contemplated. In future works, we intend to extend our research to determine the types of consortia formed in the cultivation fields and the correlation in the response capacity and development of the plant.

## Supplementary Information

**Additional file 1: Supplementary Fig. S1.** Relative abundance of species of fungi; only *Colletotrichum ignotum* showed > 1% abundance

**Additional file 2: Supplementary Fig. S2.** Relative abundance of bacterial populations; only genera with a relative abundance > 1% are shown

**Additional file 3: Supplementary Fig. S3.** Phylogenetic tree of *C. chinense* DGK. The phylogeny was reconstructed based on the alignment of the predicted protein sequences from pepper (Ca), tomato (Sol), coffee (Cc) and Arabidopsis (At). The tree was produced using the maximum likelihood method, conducting testing with 1000 bootstrap replicates, and was displayed using MEGA 6. The numbers at the nodes are the bootstrap values (> 10%), and the branch lengths from the root are displayed.

**Additional file 4: Supplementary Fig. S4.** Melting curves by *CchDGK3* and *CchPR1a*

**Additional file 5: Supplementary Fig. S5.** Melting curves by *CchEF2a3L* and *CchDGK1*.

**Additional file 6: Supplementary Fig. S6.** Melting curves by *CchPR5* and *CchNPC6*.

**Additional file 7: Supplementary Fig. S7.** Melting curves by *CchTUBa*.

**Additional file 8: Supplementary Fig. S8.** 2D-TLC-autoradiography from lipids from *C. chinense* cell cultures.

**Additional file 9: Supplementary Fig. S9.** 2D-TLC-autoradiography from lipids from *C. chinense* cell cultures infected for 6 h with a cs (1 × 10^4^).

**Additional file 10: Supplementary Fig. S10.** 2D-TLC-autoradiography from lipids from cs (1 × 10^4^).

**Additional file 11: Table S1.** Primers sets from *C. chinense* in references with *C. annuum* homologs

## Data Availability

All data generated in this study are included in the paper and in the supporting information files. Metagenomic data are available at 10.17632/mmd6f2v9z8.1. Biological materials used in the present study are available from the corresponding author upon reasonable request.

## Abbreviations

cs: conidial suspension
DAG: diacylglycerol
DAPI: 4′,6-diamidino-2-phenylindole dihydrochloride
DGK: diacylglycerol kinase
DGPP: diacylglycerol pyrophosphate
DTT: Dithiothreitol
EDTA: Ethylendiaminetetraacetic acid
FAA solution: 40% formaldehyde, 50% ethanol, 5% acetic acid, and 5% distilled water
FM4–64: (N-(3-Triethylammoniumpropyl)-4-(6-(4-(Diethylamino)Phenyl) Hexatrienyl Pyridinum Dibromide)
hai: hours after infection
HEPES: 2-[4-(2-hydroxymethyl)piperazin-1-yl] ethanesulfonic acid
5PTase: inositol polyphosphate 5-phosphatase
ITS: internal transcribed spacer
IPx: inositol polyphosphates
IPK5: inositol-pentakisphosphate 2-kinase
ITPK: inositol-tetrakisphosphate 1-kinase
JA: Jasmonic Acid
LPA: Lysophosphatidic acid
MJ: Methyl Jasmonate
MS: Murashige and Skoog medium
MTT: 3-(4,5-dimethylthiazol-2-yl)-2,5-diphenyltetrazolium bromide
NPC: nonspecific phospholipase C
PA: phosphatidic acid
PAP: phosphatidic acid phosphatase
PBS: sodium phosphate buffer
PC: phosphatidylcholine
PE: phosphatidylethanolamine
PG: phosphatidylglycerol
PI: phosphatidylinositol
PIP5K: phosphatidylinositol 4-phosphate 5 kinase
PI3K: phosphatidylinositol 3-kinase
PI4K: phosphatidylinositol 4-kinase
PI3P: phosphatidylinositol 3-phosphatase
PI5P: phosphatidylinositol 5-phosphatase.
PI4P5K: phosphatidylinositol 4-phosphate 5-kinase
PI3P5K: phosphatidylinositol 3-phosphate 5-kinase
PIP: phosphatidylinositol phosphate
PIP_2_: phosphatidylinositol bisphosphate
PIP_3_: phosphatidylinositol trisphosphate
PLA: Phospholipase A
PI-PLC: Phosphatidylinositol-specific phospholipase C
PLD: Phospholipase D
PMSF: phenylmethylsulfonyl fluoride
PS: phosphatidylserine
ROS: Reactive Oxygen Species
RT-pPCR: reverse transcription-quantitative polymerase chain reaction
SEM: Scanning Electron Microscopy
SA: Salicylic Acid
TLC: Thin Layer Chromatography
