# Supplementary figures and images for: Phospholipid signaling pathway in Capsicum chinense suspension cells as a key response to consortium infection
Source: BMC Plant Biol. 2021 Jan 25;21:62. doi: 10.1186/s12870-021-02830-z (PMC7836502; doi:10.1186/s12870-021-02830-z)

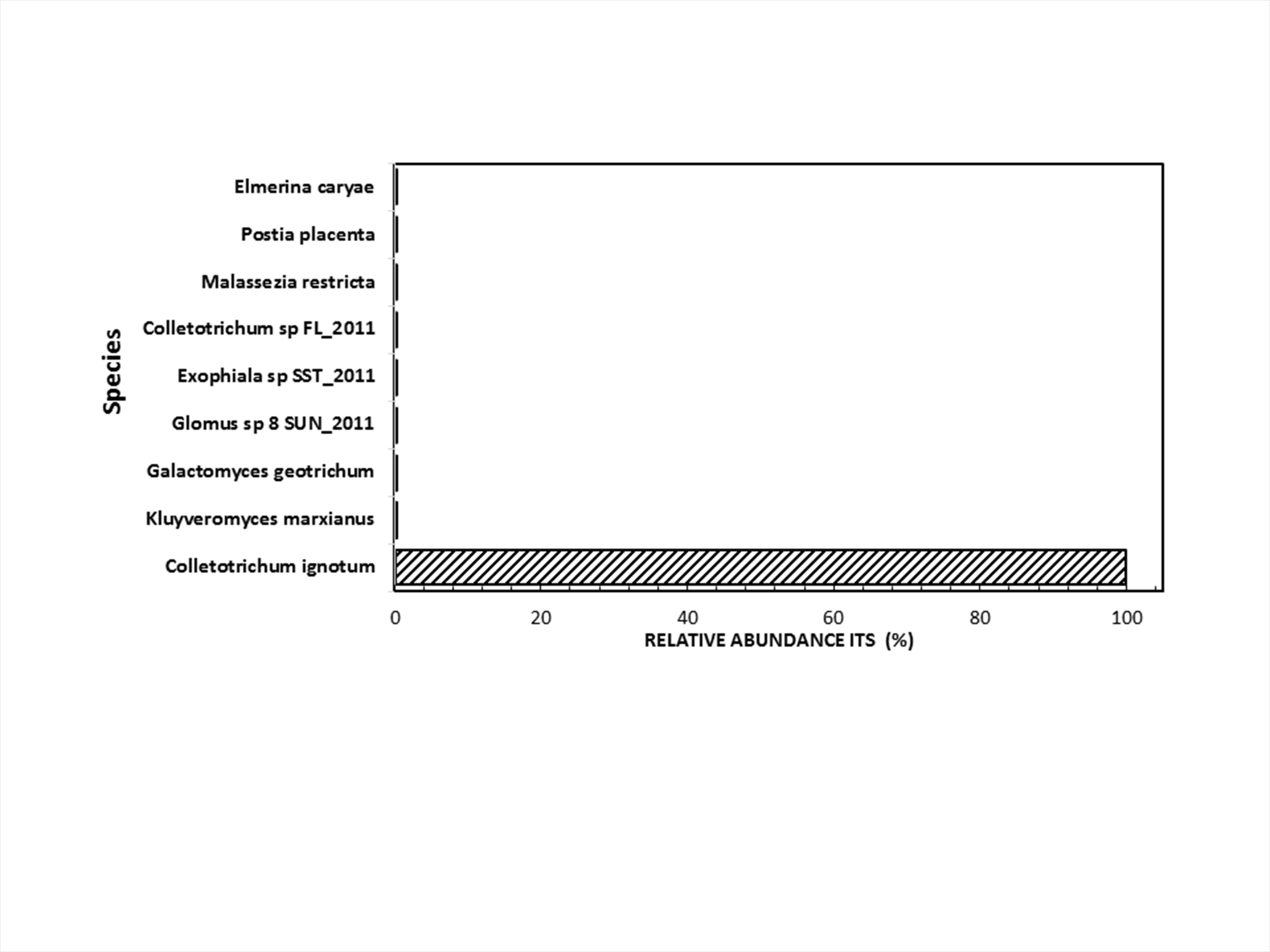

Supplement: Supplementary file 1 — Additional file 1: Supplementary Fig. S1. Relative abundance of species of fungi; only Colletotrichum ignotum showed > 1% abundance [file 12870_2021_2830_MOESM1_ESM.tif]

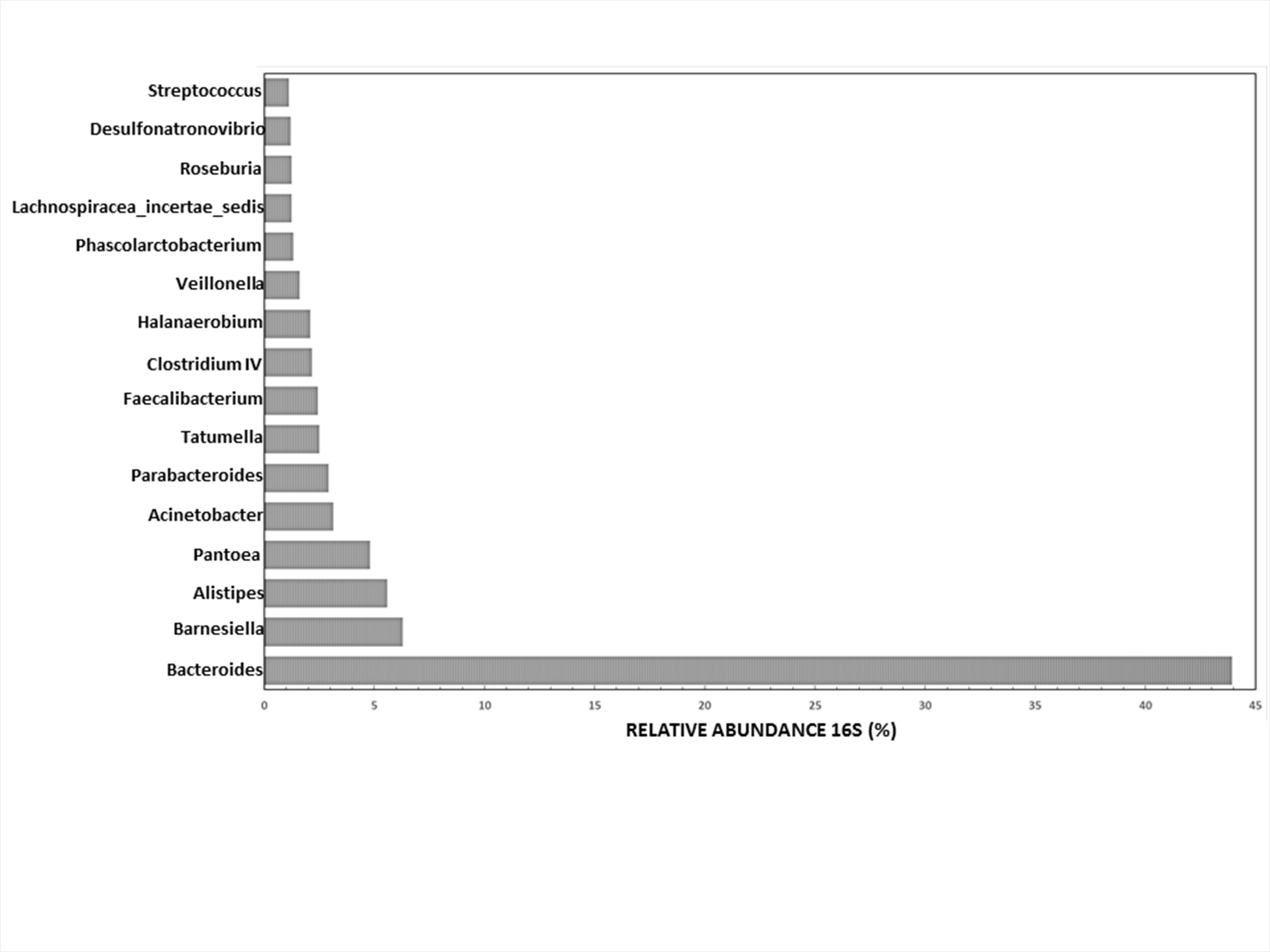

Supplement: Supplementary file 2 — Additional file 2: Supplementary Fig. S2. Relative abundance of bacterial populations; only genera with a relative abundance > 1% are shown [file 12870_2021_2830_MOESM2_ESM.tif]

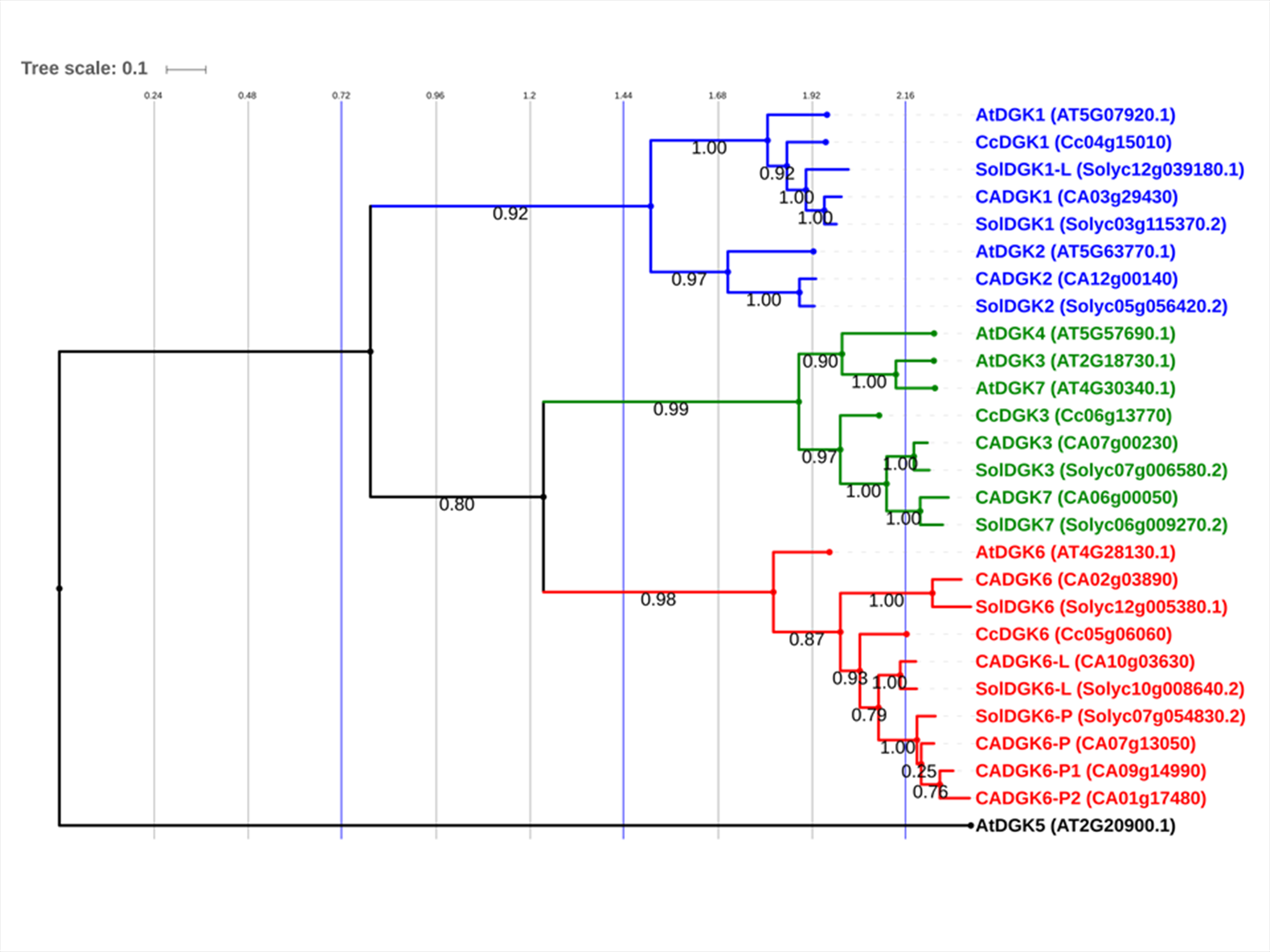

Supplement: Supplementary file 3 — Additional file 3: Supplementary Fig. S3. Phylogenetic tree of C. chinense DGK. The phylogeny was reconstructed based on the alignment of the predicted protein sequences from pepper (Ca), tomato (Sol), coffee (Cc) and Arabidopsis (At). The tree was produced using the maximum likelihood method, conducting testing with 1000 bootstrap replicates, and was displayed using MEGA 6. The numbers at the nodes are the bootstrap values (> 10%), and the branch lengths from the root are displayed. [file 12870_2021_2830_MOESM3_ESM.tif]

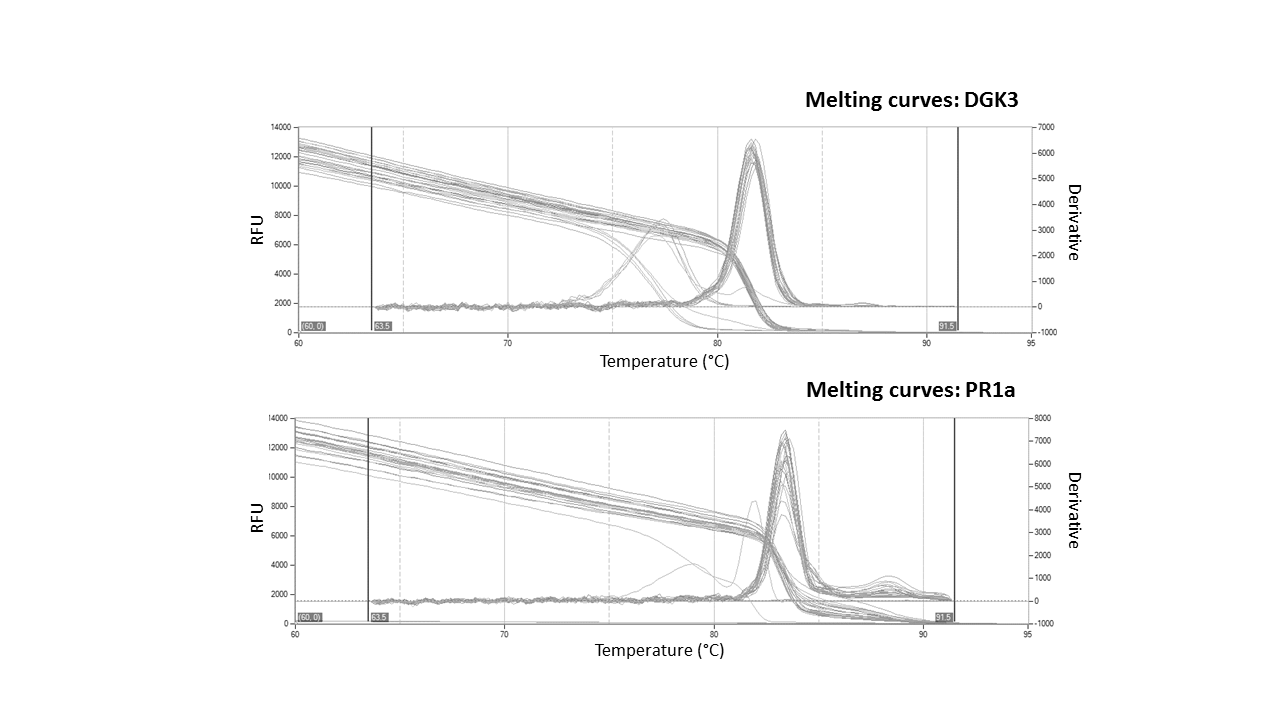

Supplement: Supplementary file 4 — Additional file 4: Supplementary Fig. S4. Melting curves by CchDGK3 and CchPR1a [file 12870_2021_2830_MOESM4_ESM.tif]

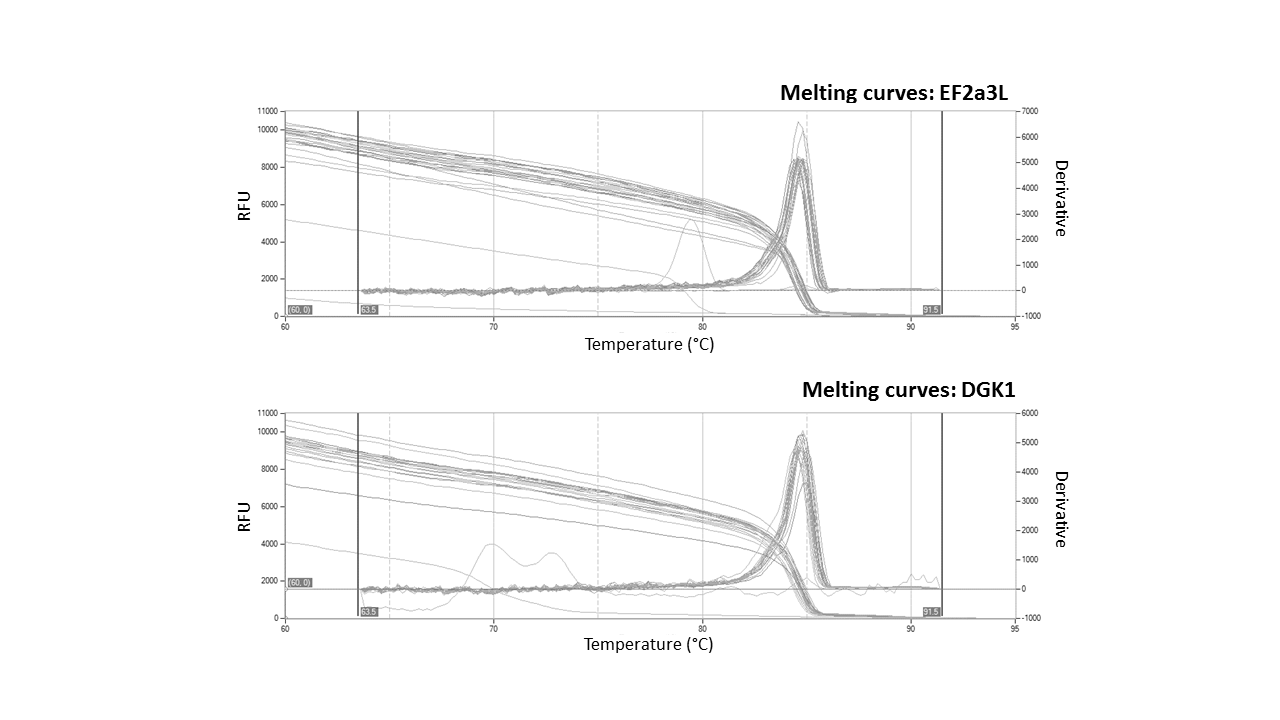

Supplement: Supplementary file 5 — Additional file 5: Supplementary Fig. S5. Melting curves by CchEF2a3L and CchDGK1. [file 12870_2021_2830_MOESM5_ESM.tif]

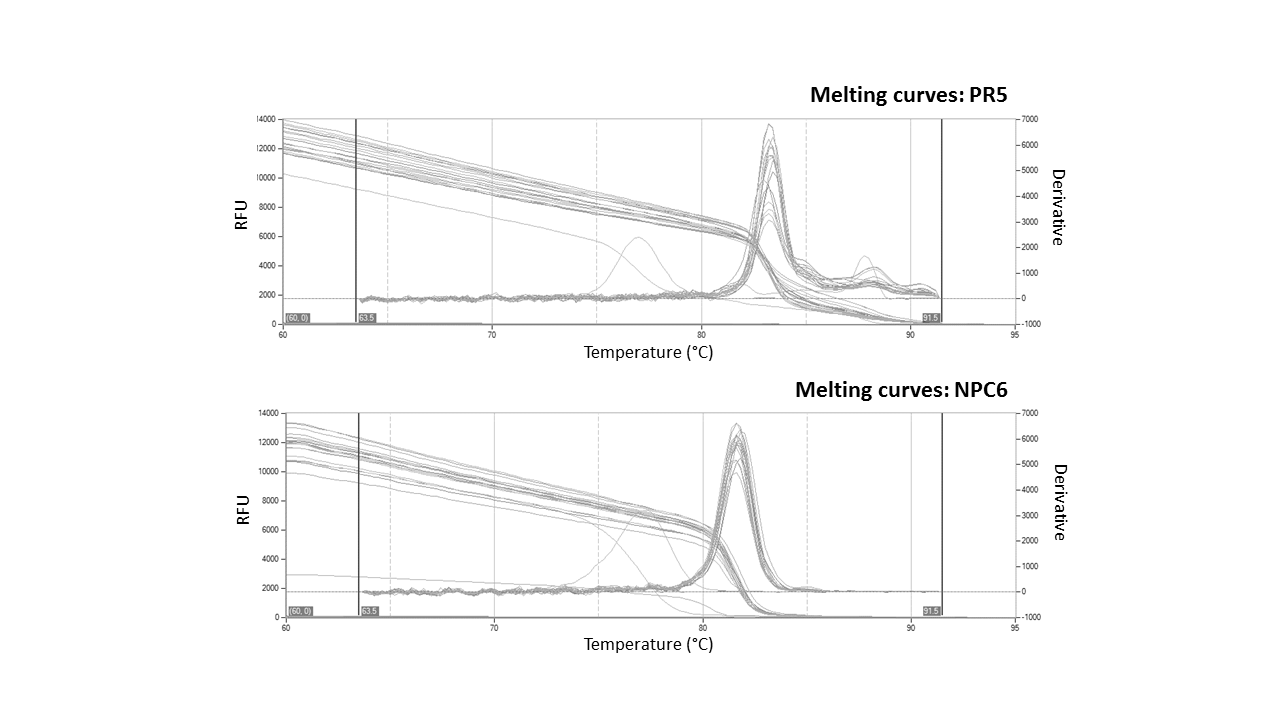

Supplement: Supplementary file 6 — Additional file 6: Supplementary Fig. S6. Melting curves by CchPR5 and CchNPC6. [file 12870_2021_2830_MOESM6_ESM.tif]

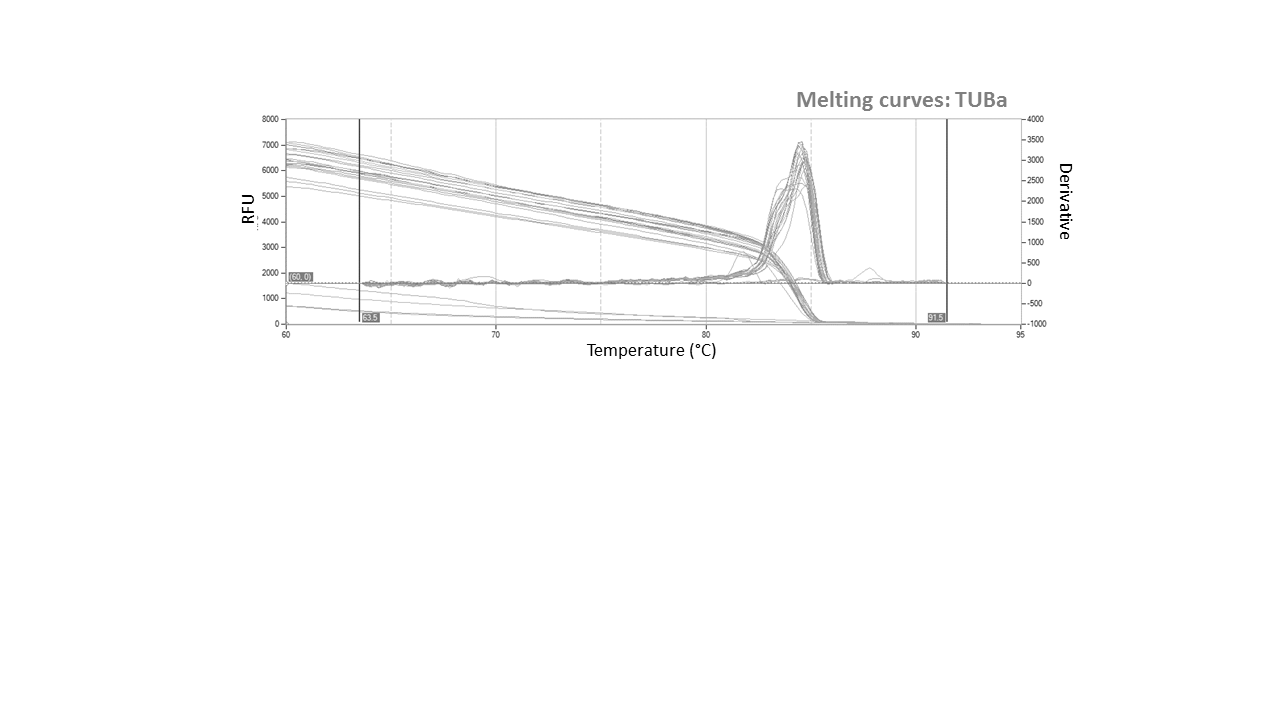

Supplement: Supplementary file 7 — Additional file 7: Supplementary Fig. S7. Melting curves by CchTUBa. [file 12870_2021_2830_MOESM7_ESM.tif]

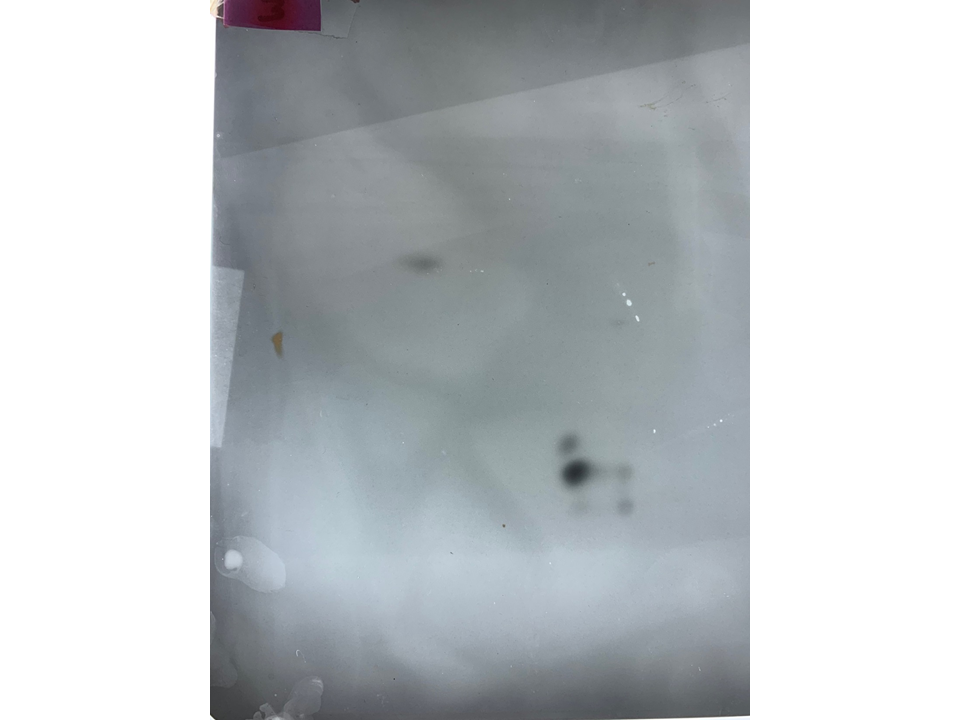

Supplement: Supplementary file 8 — Additional file 8: Supplementary Fig. S8. 2D-TLC-autoradiography from lipids from C. chinense cell cultures. [file 12870_2021_2830_MOESM8_ESM.tif]

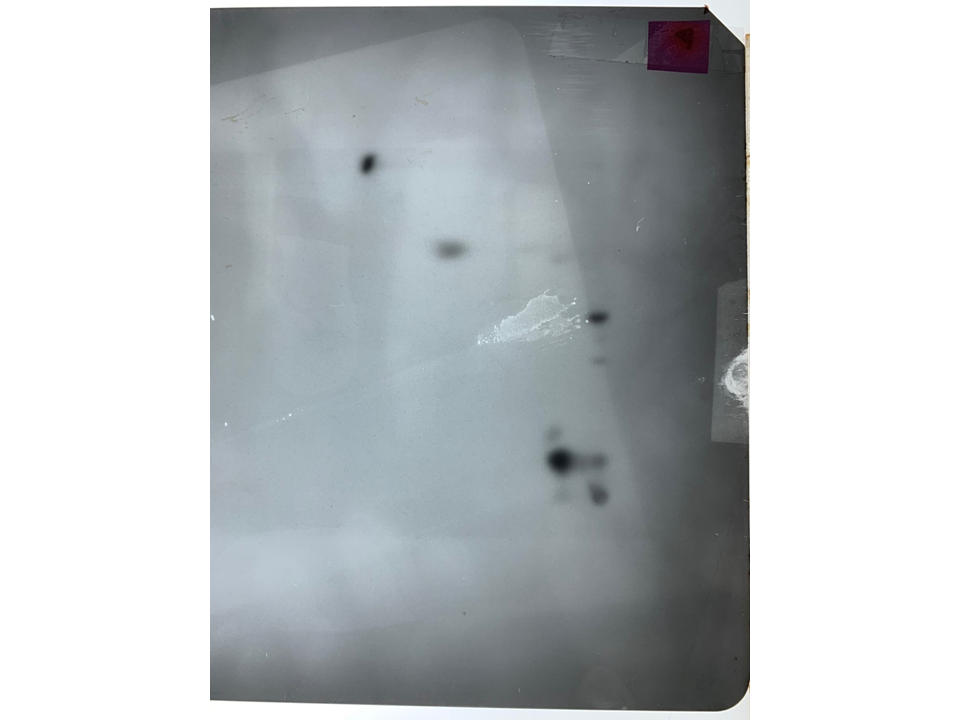

Supplement: Supplementary file 9 — Additional file 9: Supplementary Fig. S9. 2D-TLC-autoradiography from lipids from C. chinense cell cultures infected for 6 h with a cs (1 × 10^4^). [file 12870_2021_2830_MOESM9_ESM.tif]

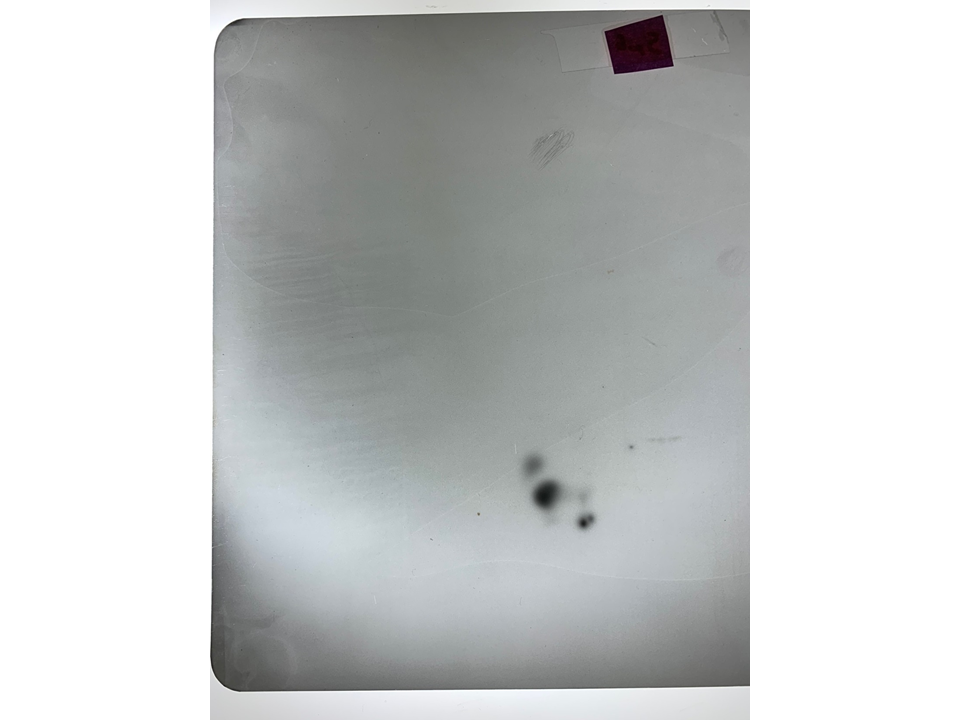

Supplement: Supplementary file 10 — Additional file 10: Supplementary Fig. S10. 2D-TLC-autoradiography from lipids from cs (1 × 10^4^). [file 12870_2021_2830_MOESM10_ESM.tif]
