# Supplementary material for: Phospholipid signaling pathway in Capsicum chinense suspension cells as a key response to consortium infection
Source: BMC Plant Biol. 2021 Jan 25;21:62. doi: 10.1186/s12870-021-02830-z (PMC7836502; doi:10.1186/s12870-021-02830-z)
Supplement: Supplementary file 11 — Additional file 11: Table S1. Primers sets from C. chinense in references with C. annuum homologs [file 12870_2021_2830_MOESM11_ESM.docx]

| **Gene** | **Primer sequence (5’---3’)** | **Gene Ref** | **Tm optimal** |
| --- | --- | --- | --- |
| **CchDGK1** | Forward: 5´-GCAAAAATCCTTCTGAGATG-3´  Reverse: 5´-GGACCTTATTCCTTGTTTTC-3 | CA03g29430 | 55 ^0^C |
| **CchDGK3** | Forward: 5´-CTTTGAATCTTCCTAGCTATGG-3´  Reverse: 5´-CATATAGGCTTCTTTCCATTCC-3 | CA07g00230 | 55 ^0^C |
| **CchNPC6** | Forward: 5´-CCTTTGCTTATGTTTTCTTGAT-3´  Reverse: 5´-ATTGATTCTTGGATTCACTGAC-3´ | CA00g75250 | 55 ^0^C |
| **CchPR1a1** | Forward: 5´-CCATTTTAGTTGCTTGTTTCCTTAC-3´  Reverse: 5´-ATTTTGTTGTGGAGAATTTTGAGC-3´ | CA01g31110 | 60 ^0^C |
| **CchPR5** | Forward: 5´-CAAATTCATGTGTTGAAGGACAATTA-3´  Reverse: 5´-CATCCAACTTTCCTTGTACTCTTC-3´ | CA01g03030 | 60 ^0^C |
| **CchEF2a3L** | Forward: 5´-GATATTCTCATGGCTACAATGCTTAA-3´  Reverse: 5´-GCATAATTTCAACAGCAGCTAAATG-3´ | CA12g21420 | 56 ^0^C |
